# Supplementary material for: BenthicNet: A global compilation of seafloor images for deep learning applications
Source: Sci Data. 2025 Feb 7;12:230. doi: 10.1038/s41597-025-04491-1 (PMC11806053; doi:10.1038/s41597-025-04491-1)
Supplement: Supplementary file 1 — Supplementary Material [file 41597_2025_4491_MOESM1_ESM.pdf]

# Supplementary Material

## A PANGAEA Search

To thoroughly search PANGAEA for seafloor imagery, we used 20 search terms with a range of synonyms for the content of interest. The PANGAEA search API is comprehensive and allows terms be combined with AND or OR operators, and negative search terms to be used. However, we could not merge all our synonyms together into a single, large query because the number of results which can be returned by one query is limited to 500 records.

The search terms used were as follows:

```
(seabed OR "sea bed" OR "sea-bed") (image OR imagery OR photo OR photograph
    OR "photo-transect" OR photoquad* OR photo-quad* OR jpg OR jpeg OR png OR tif
    OR tiff)
(seafloor OR "sea floor" OR "sea-floor") (image OR imagery OR photo OR photograph
    OR "photo-transect" OR photoquad* OR photo-quad* OR jpg OR jpeg OR png OR tif
    OR tiff)
("ocean floor" OR "ocean-floor") (image OR imagery OR photo OR photograph
    OR "photo-transect" OR photoquad* OR photo-quad* OR jpg OR jpeg OR png OR tif
    OR tiff)
underwater (habitat* OR substrate OR sediment) (image OR imagery OR photo
    OR photograph OR "photo-transect" OR photoquad* OR photo-quad* OR jpg OR jpeg
    OR png OR tif OR tiff)
benthic (image OR imagery OR photo OR photograph OR "photo-transect" OR photoquad*
    OR photo-quad* OR jpg OR jpeg OR png OR tif OR tiff)
(benthos or benthoz) (image OR imagery OR photo OR photograph OR "photo-transect"
    OR photoquad* OR photo-quad* OR jpg OR jpeg OR png OR tif OR tiff)
(coral OR reef OR seagrass OR "sea grass") (image OR imagery OR photo OR photograph
    OR "photo-transect" OR photoquad* OR photo-quad* OR jpg OR jpeg OR png OR tif
    OR tiff)
(auv OR rov OR uuv OR "underwater vehicle") (image OR imagery OR photo OR photograph
    OR "photo-transect" OR photoquad* OR photo-quad* OR jpg OR jpeg OR png OR tif
    OR tiff)
benthoscape habitat* image
benthoscape habitat* imagery
benthoscape habitat* photo
benthoscape habitat* photograph
benthoscape habitat* ("photo-transect" OR photoquad* OR photo-quad*)
benthoscape habitat* (jpg OR jpeg OR png OR tif OR tiff)
benthoscape image
benthoscape imagery
benthoscape photo
benthoscape photograph
benthoscape ("photo-transect" OR photoquad* OR photo-quad*)
benthoscape (jpg OR jpeg OR png OR tif OR tiff)
```

Each search term was prefixed with a set of negative search terms to remove false positives, given as follows

```
-microscop? -"Meteorological observations" -topsoil -soil -sky
    -"wind vector" -"wind stress" -"vertical profile" -"vertical distribution"
```

The full code for our PANGAEA search is publicly available at https://github.com/DalhousieAI/pangaea-downloader.

## B FathomNet Python API Code

We retrieved the full set of images on FathomNet by using the FathomNet API from the fathonnet-py Python package as follows.

```python
import fathomnet.api.images
import pandas as pd

keys = ["url", "uuid", "timestamp", "latitude", "longitude"]

records = []
for submitter in fathomnet.api.images.find_distinct_submitter():
    for image in fathomnet.api.images.find_by_contributors_email(submitter):
        records.append({k: getattr(image, k) for k in keys})

df = pd.DataFrame.from_records(records)
df.drop_duplicates(subset="url", inplace=True)
```
